# Effects of a ‘Baby-Friendly Hospital Initiative’ on exclusive breastfeeding rates at a private hospital in Lebanon: an interrupted time series analysis

**DOI:** 10.1186/s12884-021-03816-3

**Published:** 2021-05-19

**Authors:** Adrienne Clermont, Josianne El Gemayel, Rola Hammoud, Jiangxia Wang, Hortenzia Beciu, Mona Sinno, Wilma Berends, Nadine Rosenblum, Jessica L. Bienstock, Kristen Byrnes, Roger Samuels

**Affiliations:** 1 Department of International Health, Johns Hopkins Bloomberg School of Public Health, Baltimore, MD USA; 2 Department of Obstetrics and Gynecology, Clemenceau Medical Center, Clemenceau St, Beirut, Lebanon; 3 Department of Quality Management, Clemenceau Medical Center, Clemenceau St, Beirut, Lebanon; 4 Department of Biostatistics, Johns Hopkins Biostatistics Center, Johns Hopkins Bloomberg School of Public Health, Baltimore, MD USA; 5 Johns Hopkins Medicine International, Baltimore, MD USA; 6 Department of Gynecology and Obstetrics, Johns Hopkins University School of Medicine, Baltimore, MD USA; 7 Department of Medicine, Division of Pulmonary/Critical Care Medicine, Johns Hopkins University School of Medicine, Baltimore, MD USA

**Keywords:** Breastfeeding, Lebanon, Maternity Care, Neonatal care, Quality Improvement

## Abstract

**Background:**

Exclusive breastfeeding (EBF) through six months of age has been scientifically validated as having a wide range of benefits, but remains infrequent in many countries. The WHO/UNICEF Baby-Friendly Hospital Initiative (BFHI) is one approach to improve EBF rates.

**Methods:**

This study documents the implementation of BFHI at Clemenceau Medical Center (CMC), a private hospital in Lebanon, and analyzes data on EBF practices among CMC’s patients before, during, and after the implementation period. The process of launching the BFHI at CMC is discussed from the perspective of key stakeholders using the SQUIRE guidelines for reporting on quality improvement initiatives. As an objective measure of the program’s impact, 2,002 live births from July 2015 to February 2018 were included in an interrupted time series analysis measuring the rates of EBF at discharge prior to, during, and following the bundle of BFHI interventions.

**Results:**

The steps necessary to bring CMC in line with the BFHI standards were implemented during the period between November 2015 and February 2016. These steps can be grouped into three phases: updates to hospital policies and infrastructure (Phase 1); changes to healthcare staff practices (Phase 2); and improvements in patient education (Phase 3). The baseline percentage of EBF was 2.4 % of all live births. Following the BFHI intervention, the observed monthly change in EBF in the “Follow-Up” period (i.e., the 24 months following Phases 1–3) was significantly increased relative to the baseline period (+ 2.0 % points per month, *p* = 0.006). Overall, the observed rate of EBF at hospital discharge increased from 2.4 to 49.0 % of all live births from the first to the final month of recorded data.

**Conclusions:**

Meeting the BFHI standards is a complex process for a health facility, requiring changes to policies, practices, and infrastructure. Despite many challenges, the results of the interrupted time series analysis indicate that the BFHI reforms were successful in increasing the EBF rate among CMC’s patients and sustaining that rate over time. These results further support the importance of the hospital environment and health provider practices in breastfeeding promotion, ultimately improving the health, growth, and development of newborns.

**Supplementary Information:**

The online version contains supplementary material available at 10.1186/s12884-021-03816-3.

## Background

### Problem description and available knowledge

The practice of exclusive breastfeeding (EBF) of newborn infants through six months of age has been validated as having a wide range of health benefits for both baby and mother [1, 2], as well as broader economic and environmental benefits for society [3]. It is recommended as the standard for infant feeding by the World Health Organization (WHO) [4], the American Academy of Pediatrics [1], the American College of Obstetricians and Gynecologists [5], and the Lebanese Ministry of Health [6]. However, despite knowledge of these benefits within the scientific and clinical community and nearly three decades of initiatives to promote breastfeeding, EBF rates remain low in many areas of the world [3]. A variety of factors – including sociocultural attitudes, workplace and government policies, marketing of breastmilk substitutes, and entrenched clinical practices – contribute to lowered levels of EBF, particularly in higher-income populations and countries [3]. Accordingly, the translation of EBF principles into general healthcare practice represents an important and evolving target for structured efforts at behavioral change within healthcare organizations.

Lebanon is an upper-middle-income country that is known to have low levels of sustained EBF. Although more recent nationally representative survey data are not available, a 2006 national survey found that while almost all mothers (95.4 %) initiated breastfeeding, by one month, only 52.4 % of babies were exclusively breastfed [7]. This declined further to 23.4 % at four months and only 10.1 % at six months. EBF was found to be inversely associated with urban residence and educational level of the mother. A number of studies have examined the causes of low EBF rates in Lebanon, and have identified hospital-related factors (such as lack of rooming-in) [7]; maternal employment and related barriers [8–11]; negative perceptions of breastfeeding (fear of weight gain and breast sagging) and experiences of breastfeeding (insufficient milk, pain, sleep deprivation) [9]; cultural beliefs around “bad milk” and potential harm to the infant [12]; and lack of government policy engagement to promote breastfeeding [9, 13].

Hospitals and healthcare providers are an important avenue for the promotion of EBF to new mothers. The Baby-Friendly Hospital Initiative (BFHI) was developed by the WHO and UNICEF in 1991 (updated in 2009 and 2018) as a “global effort to implement practices that protect, promote, and support breastfeeding” [14–16]. It is comprised of a 10-step, evidence-based program to improve policies, training, and practices at hospitals and clinics providing maternity care. Since its inception, the BFHI program has been implemented in over 20,000 health facilities in 152 countries worldwide [14, 17]. One recent systematic review found a positive relationship between implementation of the BFHI program and improved breastfeeding outcomes, with a dose-response between the number of BFHI steps to which women were exposed and the likelihood of EBF [18], while another systematic review found the heterogeneity in study designs and settings too high to draw an overall conclusion on BFHI’s effectiveness [19]. Recent critical re-evaluations of BFHI have suggested that focusing on specific evidence-based interventions for breastfeeding may be more important than adherence to the overall package or the BFHI certification itself [20]. In the Middle East region in particular, studies have documented the effectiveness of BFHI implementation in hospitals in Saudi Arabia [21] and the United Arab Emirates [22, 23].

### Rationale and specific aims

This study describes the experience of Clemenceau Medical Center (CMC), located in Beirut, Lebanon, with the implementation of the BFHI program in 2015. It analyzes data on EBF practices from 2,002 live births from July 2015 to February 2018 at CMC. This paper has two objectives: first, to describe the development and execution of an EBF program at CMC in the context of the BFHI model, using the SQUIRE guidelines for reporting quality improvement initiatives [24]; and second, to conduct a quantitative statistical evaluation of the impact of BFHI practices on EBF rates at discharge using interrupted time series analysis.

## Methods

### Setting and context

CMC is a 158-bed Joint Commission International (JCI)-accredited private hospital in Beirut, Lebanon, with approximately 750 deliveries annually. CMC has an active affiliation with Johns Hopkins Medicine International (JHI), through which a number of collaborations have been undertaken to promote quality and patient safety improvement, education, and strategic planning initiatives across several of CMC’s hospital divisions. One such collaborative initiative, launched at CMC in 2015, was an effort to establish the standards outlined in the BFHI in order to respond to the challenges of low regional EBF rates.

### Intervention

Beginning in October 2015, medical and administrative staff at CMC, with support from partners at JHI, implemented a series of actions to more systematically align CMC’s policies and practices with those proposed by the BFHI. The process was spearheaded by the office of the Chief Quality Officer at CMC and included a core team of midwives, nurses, and physicians, as well as technical support from Johns Hopkins subject matter experts in obstetrics, lactation nursing, and project management. The implemented reforms can be grouped into three phases: updates to hospital policies and infrastructure (Phase 1); changes to healthcare staff practices (Phase 2); and improvements in patient education (Phase 3). The key interventions are summarized in Table 1, and a more detailed list of interventions with a timeline, grouped according to the “10 Steps” of the BFHI process, is available in Additional file 1.

**Table 1:** Summary of key interventions in BFHI process at CMC

Category	Key interventions
Phase 1: Hospital policies and infrastructure	• Completion of WHO self-assessment tool • Revision of existing newborn policy • Drafting of new policies on EBF, skin-to-skin contact, and rooming-in • Revision of existing “no formula on the wards” policy • Construction of a private breastfeeding room for consultations and patient use
Phase 2: Healthcare staff practices	• Formation of an EBF team to lead BFHI implementation process • Incorporation of EBF topics into institutional training for new hires and current staff • Implementation of rooming-in and skin-to-skin practices with patients • Allocation of expert lactation consultant to supervise midwife/nurse team • Addition of newborn flow sheet to electronic medical record to document feedings • Requirement of medical order by pediatrician for an infant to receive formula
Phase 3: Patient education	• Addition of EBF session into prenatal education classes for expecting mothers • Provision of informational materials on breastfeeding and infant care at discharge • Availability of lactation consultant for post-discharge consultations five days a week • Creation of breastfeeding support phone line for questions 24 h a day

The first phase of interventions pertained to aligning CMC hospital policies with the model outlined by the BFHI standards proposed by the WHO and UNICEF. After completing the “WHO Self-Assessment Tool,” which is intended to assess a health facility’s status prior to instituting BFHI, the team at CMC, in collaboration with the JHI team, revised existing policies and drafted several new ones to reflect a commitment to the adoption of evidence-based BFHI practices across the institution. This ensured the buy-in of the hospital leadership and provided a policy framework for the subsequent adjustment of practices. In parallel, infrastructure improvements intended to facilitate breastfeeding were implemented: the “nursery” area was closed for renovations and subsequently reopened with its name changed to “breastfeeding room” in order to encourage rooming-in (see below).

The second phase encompassed the implementation of programs designed to improve healthcare staff practices. Led by a newly created “EBF Team” (composed of midwives, nurse educators, and physicians) that championed the process, a variety of changes were instituted that made breastfeeding an integral part of the prenatal and postnatal care teams’ work. Per WHO guidelines, EBF-related topics were incorporated into training for all new and existing CMC staff (clinical and non-clinical), and a lactation consultant with Masters-level training was delegated to supervise the midwives and nurses on the maternity ward. Several practices with a strong evidence base for improving breastfeeding practices, such as early skin-to-skin contact [25], rooming-in [26], and allowing formula use only with a medical order from the pediatrician, were instituted. Finally, CMC’s electronic medical record system was supplemented with an electronic version of the “newborn flow sheet,” which had previously been hard-copy only. This aimed specifically to track feeding practices and to collect all data for better analysis.

The third phase of the intervention centered on patient education, a crucial component for behavior change given the baseline extremely low rates of EBF among the patient population. This phase included the addition of breastfeeding information into CMC’s prenatal education classes; creation of promotional materials inside patient rooms (e.g., as inspirational messages on bathroom mirrors) and on take-home items (e.g., babies’ bibs and hats); better documentation and standardization of prenatal and postnatal breastfeeding consultations by healthcare providers; detailed charting of breastfeeding practices at discharge; and the establishment of in-person and phone-based resources for new mothers post-discharge. Lastly, promotional campaigns at the hospital and online (via CMC’s website and Facebook page) have sought to raise the profile of breastfeeding among patients and providers.

### Data collection and measures

Breastfeeding data from 2,002 newborns delivered from July 2015 to February 2018 at CMC were examined to determine the overall impact of the collaborative BFHI process on monthly EBF rates. This period includes four months prior to intervention (“Lead-In”), the four-month window encompassing the bulk of the intervention period (“Intervention”), and 24 months of post-intervention follow-up (“Follow-Up”). The EBF data are drawn from de-identified, aggregated hospital tracking data that include all live births during this time period.

For the purpose of this analysis, “exclusively breastfed” implies that mother and infant completed their entire inpatient stay (from birth to discharge, typically 2–4 days after birth) without the use of supplemental formula or other non-breastmilk liquids such as water or tea. Accordingly, monthly EBF rates were calculated as the number of “exclusively breastfed” infants divided by the number of total live births. Exclusion criteria were determined by CMC pediatricians and included infants managed in the intensive care setting, twin births, and infants for whom formula use was medically indicated.

### Data analysis

Analysis of EBF data was performed via interrupted time series analysis with multiple treatment periods following a first-order autoregressive process. The ITSA command suite in STATA Version 15 software was utilized [27, 28]. For the purposes of this data analysis, the “Intervention” was defined as the period between November 1, 2015, and February 29, 2016, during which the majority of the Phase 1–3 activities occurred.

### Ethical considerations

EBF and live birth rates were collected via de-identified hospital reporting data, and independently validated and audited by a third party through random, direct, on-site chart reviews. Data were stored in a HIPAA-compliant portal. Data collection procedures and protocols were reviewed by the Johns Hopkins Medicine Office of Human Subjects Research Institutional Review Board (application #IRB00155989), and determined to not constitute human subjects research under DHHS or FDA regulations. Confirmation was obtained from Clemenceau Medical Center that this study did not require formal review by its Institutional Review Board as it did not constitute human subjects research. No additional administrative permissions or licenses were required to access the de-identified, aggregated data used in this study.

## Results

**Fig. 1:**
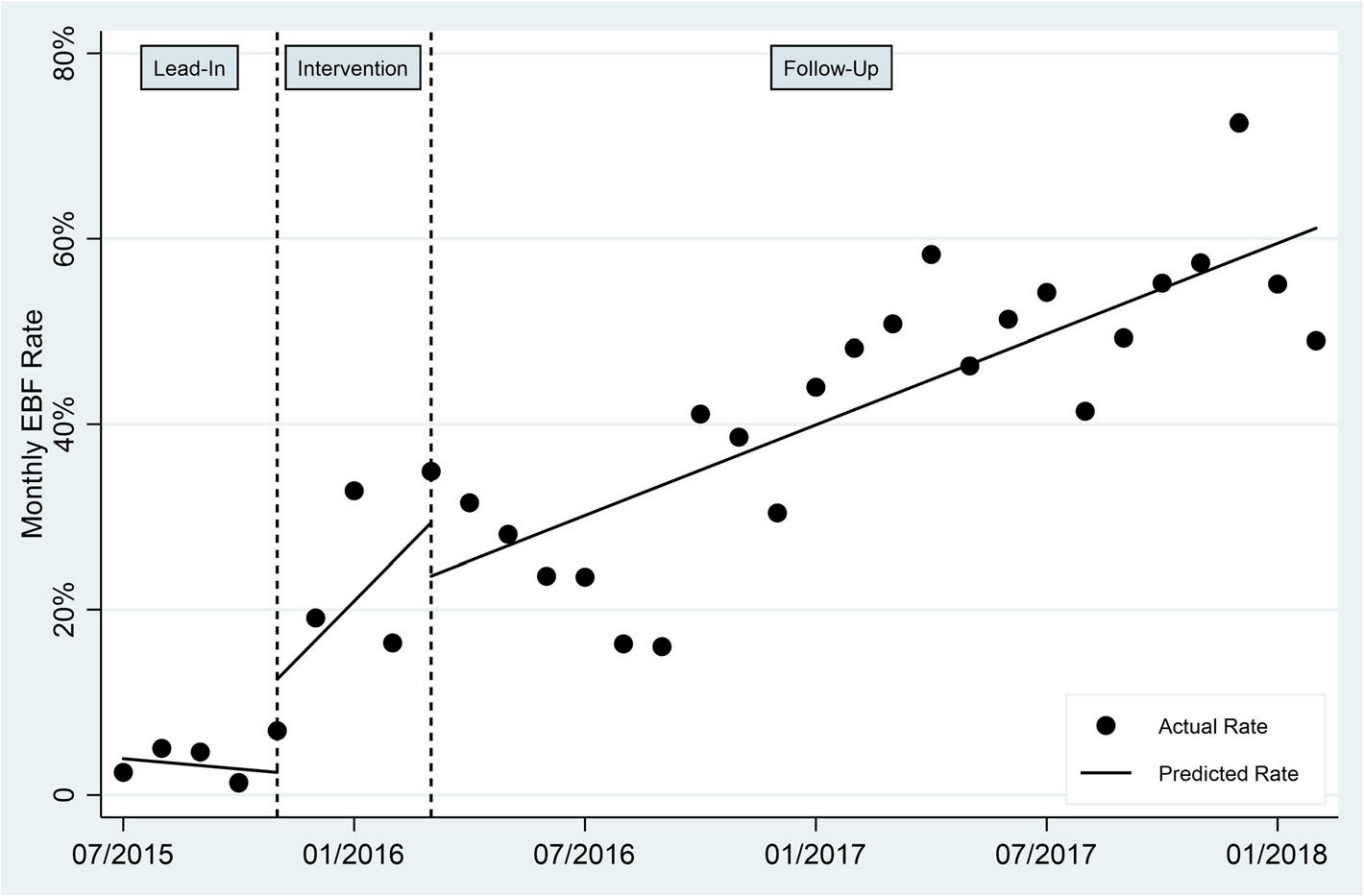
Interrupted Time Series Analysis of Exclusive Breastfeeding Rates at CMC. Note: The dashed vertical lines represent the time period from November 1, 2015, to February 29, 2016 during which the majority of BFHI interventions occurred (i.e., Phases 1–3)

The results of the interrupted time series analysis are shown in Fig. 1. In the first month for which data were collected (July 2015), the EBF compliance rate was 2.40 %. The baseline mean EBF compliance rate during the “Lead-In” period (July 2015 through October 2015, prior to most BFHI activities) was 3.33 % (95 % confidence interval [CI]: 1.60, 5.06). There was a non-significant decreasing trend in the EBF compliance rate during the “Lead-In” period of -0.37 % points per month (*p* = 0.54, 95 % CI: −1.58, 0.84).

During the “Intervention” period (November 2015 through February 2016, in which the bulk of BFHI interventions occurred), the monthly change in EBF compliance was non-significantly greater than the “Lead-In” (pre-intervention) trend by + 4.59 % points per month (*p* = 0.16, 95 % CI: -2.00, 11.17). Mean EBF compliance during the “Intervention” period was 18.80 % (95 % CI: 8.30, 29.30).

During the “Follow-Up” period (March 2016 through February 2018, after the bulk of BFHI interventions were completed), the monthly rate of change of EBF was lower relative to the “Intervention” period trend by -2.59 % points per month (*p* = 0.39, 95 % CI = -8.67, 3.49) but was statistically significantly greater than the trend during the baseline “Lead-In” period by + 2.00 % points per month (*p* < 0.006, 95 % CI: 0.61, 3.39). The overall mean EBF compliance rate during the “Follow-Up” period was 42.38 % (95 % CI: 36.62, 48.14). In the final month for which data were collected (February 2018), the EBF compliance rate was 49.00 %.

Hence, in considering the overall impact of the BFHI process (comparing the post-intervention “Follow-Up” period to the pre-intervention “Lead-In” period), the intervention resulted in a statistically significant increase in the monthly rate of change in EBF compliance of + 2.00 % points per month and an overall increase in EBF compliance, from 2.40 % in the first month of data collection to 49.00 % in the last month of data collection. Graphical and numerical comparisons of practices in the three periods are presented in Fig. 1; Table 2.

**Table 2:** Comparison of exclusive breastfeeding compliance before, during, and after a series of institutional interventions aimed at promoting Baby-Friendly Hospital practices

**Outcome measure**	“Lead-in” (*n* = 326 births)	“Intervention” (Phases 1-3)(*n*= 245 births)	“Follow-up” (*n* = 1,431 births)
**Mean EBF Rate (95 % confidence interval)**	3.33 % (1.60, 5.06)	18.80 % (8.30, 29.30)	42.38 % (36.62, 48.14)
**Monthly change in EBF rate (95 % confidence interval)**	-0.37 % points (-1.58, 0.84)	+ 4.22 % points (-1.95, 2.31)	+ 1.63 % points (0.96, 2.31)
**Difference in monthly change in EBF rate (95 % confidence interval)**	*Reference*	4.59 (-2.00, 11.2; *p* = 0.164)	2.00 (0.61, 3.39; *p* = 0.006)

## Discussion

This study uses an interrupted time series methodology to demonstrate the impact of implementing the BFHI package among an initially EBF-averse patient population. The absolute rate of EBF at hospital discharge increased from 2.4 to 49.0 % from the first month to the final month of data recorded, and the above analysis conveys the improved trends in EBF practices over time. This analysis showed overall increases in the adoption of EBF during both the “Intervention” and “Follow-Up” periods, as well as a statistically significant change in the monthly increase in EBF adoption when compared to baseline. Moreover, these observed behavioral trends in the adoption of EBF practices persisted up to two years following the initial intervention period.

Implementation of the full BFHI package is a complex intervention. Each of the WHO/UNICEF-validated “10 Steps” involves multiple discrete actions and changes that require buy-in from hospital administration, healthcare staff, and patients. Using Powell et al.’s framework from the implementation science literature, this process included implementation strategies related to planning, education, restructuring, and quality management [29]. A timeline of when specific interventions were implemented is available in Additional file 1; however, many of these changes were gradual, and the effect of the BFHI package was likely cumulative over time.

From the CMC team’s perspective, a key factor in the successful implementation of BFHI and the resulting increase in EBF rates was the engagement of a core team of care providers. This multidisciplinary team included midwives, nurse educators, pediatricians, and obstetricians. They served as “champions” for the BFHI process and led the process of policy revision and implementation of new procedures. The CMC team cited pediatricians as very influential in women’s decisions to breastfeed, a finding that is supported by several previous studies in Lebanon [7, 11]. Hospital administration and management were also engaged in the BFHI process, allowing for institution-wide reforms and unified messaging. Positive feedback from future mothers and their families regarding CMC’s BFHI improvements and the surrounding promotional campaign has provided the momentum for the process to continue.

Several challenges arose during BFHI implementation, many of which reflect cultural barriers to breastfeeding that persist in Lebanon. For practices such as skin-to-skin contact after birth, adoption of such practices by women occurred quickly, demonstrating that successful behavior change is possible. However, many CMC patients, family members, and even occasionally outpatient healthcare providers still expressed doubt that breastmilk is nutritionally sufficient for newborns. Some mothers also resisted the rooming-in policy, expressing the need to rest while their infant is cared for in a nursery. The CMC team addressed these challenges by adopting patient-centered communication techniques and focusing on the benefits of breastfeeding for both mother and newborn. Women were empowered to make their own decisions and were supported accordingly. Continuing educational outreach, particularly on the sufficiency of breastmilk for infant nutrition, will be important to address these challenges and further increase EBF rates in the future.

Another challenge in implementing this initiative is reflective of the fact that CMC employs a combination of full-time clinicians in addition to part-time providers from the community. For those part-time providers (who primarily practice outside the hospital but bring their patients to CMC for delivery), it has been more difficult to become engaged in the Phase 2 activities aimed at changing clinical practices, and accordingly, EBF adoption has been slower in this group. Further targeted outreach to these providers is planned.

Finally, an ongoing audit of the long-term breastfeeding practices of new mothers in the outpatient setting (i.e., following discharge from the hospital) is still actively being developed. Although our study and others show that BFHI can be successful in increasing EBF in early infancy, maintaining EBF through the recommended six months of age remains a challenge [7, 30]. For example, in their BFHI trial in Saudi Arabia, Mosher et al. showed a significant decrease in EBF rates at six months among both the intervention (BFHI hospital) and control (non-BFHI hospital) groups [21]. Systematic reviews have shown the importance of ongoing postpartum support to mothers for EBF continuation [18, 31], corresponding to Step 10 in the BFHI package. In Lebanon, a previous study has shown the positive impact of a telephone support hotline on EBF rates [8], an encouraging result for the hotline set up by CMC as a part of this study. A recently-completed randomized controlled trial at two other hospitals in Lebanon has shown the effectiveness of a comprehensive package of professional and peer postpartum support on EBF knowledge and practice [30, 32]. Looking forward, CMC will evaluate such initiatives for their utility in maintaining breastfeeding practices after new mothers have left the inpatient setting.

### Strengths and limitations

This study has a number of important strengths. Although data were analyzed retrospectively, they were collected continuously throughout the initiative, which allows for evaluating trends over time rather than mere pre/post comparisons. Interrupted time series is a strong quasi-randomized study design when the use of a control group is not feasible [33, 34]. In addition, this study’s use of patient data allowed for an objective measure (as recorded by healthcare providers) of EBF practice, compared to mothers’ self-report in post-discharge surveys used in other studies [21]. The EBF data have been strictly audited as a part of CMC’s partnership with JHI and validated by the quality department at CMC. Finally, the study sample size was large, with 2,002 live births included.

It is also important to acknowledge several limitations of this study. It was conducted retrospectively and in a single center, meaning that no control group was present; however, the interrupted time series analysis is a methodologically appropriate means for addressing this limitation [33]. In addition, because de-identified hospital reporting data were used, we were not able to measure demographic information of mothers or infants and account for any demographic shifts over time. This is an important avenue for further research, which could include prospective enrollment of participants and collection of individual-level data, in order to analyze trends among specific subgroups of mothers. Finally, our outcome measure in the evaluation of this program is the EBF rate at discharge (typically 2–4 days after birth), which, although clearly associated with EBF rates at one and six months of age [7], is admittedly a surrogate marker for such practices. Future studies at CMC should include post-discharge follow-up through six months of age, to allow for comparison with national EBF trends.

## Conclusions

In Lebanon and elsewhere, hospital-based prenatal and early postnatal interactions are a crucial entry point for behavior change regarding breastfeeding [7, 20]. Although the impact of BFHI certification in and of itself has been questioned, it is clear that specific evidence-based interventions within the BFHI package can lead to meaningful improvements in breastfeeding practice [20, 35]. The results of our study further support the importance of the hospital environment in improving EBF rates, particularly in a cultural and clinical setting with low baseline compliance.

The CMC experience highlights several important factors for healthcare institutions considering the implementation of BFHI standards. First is the use of a multidisciplinary team of care providers who can champion institutional baby-friendly practices while also dispelling misconceptions through sustained educational outreach to clinical providers and hospital staff. Next, clinician-led promotional efforts centered on prenatal education on EBF for future mothers and their families, as well as regular tracking of data to measure the adoption of breastfeeding practices in new mothers, is critical. Finally, the creation of programs designed to educate and support new mothers in sustaining such breastfeeding practices in the outpatient setting is an essential component. Upholding the principles outlined in the BFHI is a complex process that involves changes to hospital policies, staff practices, and physical infrastructure. However, with an appropriate institutional commitment, as well as staff engagement across many levels of patient care, it can be an achievable goal, leading to meaningful improvements in breastfeeding practice. 

## Supplementary information

Additional file 1 Timeline of Interventions for Baby-Friendly Hospital Initiative (BFHI) Implementation at Clemenceau Medical Center, Beirut, Lebanon

## Data Availability

The datasets used and/or analyzed during the current study are available from the corresponding author on reasonable request.

## Abbreviations

EBF: Exclusive breastfeeding
WHO: World Health Organization
BFHI: Baby-Friendly Hospital Initiative
CMC: Clemenceau Medical Center
JCI: Joint Commission International
JHI: Johns Hopkins Medicine International
