# Supplementary material for: Effects of a ‘Baby-Friendly Hospital Initiative’ on exclusive breastfeeding rates at a private hospital in Lebanon: an interrupted time series analysis
Source: BMC Pregnancy Childbirth. 2021 May 19;21:385. doi: 10.1186/s12884-021-03816-3 (PMC8132377; doi:10.1186/s12884-021-03816-3)
Supplement: Supplementary file 1 — Timeline of Interventions for Baby-Friendly Hospital Initiative (BFHI) Implementation at Clemenceau Medical Center, Beirut, Lebanon [file 12884_2021_3816_MOESM1_ESM.pdf]

Timeline of Interventions for Baby-Friendly Hospital Initiative (BFHI) Implementation at Clemenceau Medical Center, Beirut, Lebanon

Action Item	Implementation Time Period *(start date for ongoing activities)*	Notes
STEP 1. Have a written breastfeeding policy that is routinely communicated to all healthcare staff.		
Completion of WHO self-assessment tool	Aug 2015 – Oct 2015	This tool (developed by WHO and modified by JHI) is intended to assess a health facility's status prior to instituting BFHI policies and procedures. The self-assessment process at CMC was followed by videoconference discussions with the JHI EBF team (midwives and head nurses) to share learnings from the Hopkins experience of becoming BFHI certified.
Formation of the Exclusive Breastfeeding (EBF) Team	Oct 2015 – Nov 2015	This team is led by a health promotion midwife and includes representatives from the nursing administration, quality department, dietary department, NICU/pediatric unit, and maternity unit. Members include nurse educators, a pediatrician, an obstetrician, and midwives.
Creation of new EBF Policy	Oct 2015 – Apr 2016	This policy covers topics including breastfeeding counseling and promotion, contraindications to breastfeeding, and milk storage guidelines.
Revision of existing Newborn Policy	Nov 2015 – Jan 2016	This policy covers topics including assessment, feeding, bathing, and labs.
STEP 2. Train all health care staff in skills necessary to implement the policy.		
All new hires (clinical and administrative) receive training on exclusive breastfeeding policies and practices	Dec 2015	Information on EBF has been incorporated into the general orientation session for all new CMC employees and in the annual mandatory training for all staff.
Training for current physicians, nurses, nurse assistants, dietitians, midwives, and housekeeping staff	Jan 2016	This included the design, development, and implementation of a new 6-hour one-on-one training for nurse assistants led by a lactation consultant (outline available upon request), as well as implementation of a validated 20-hour online BFHI curriculum for midwives, NICU nurses, and dieticians (https://step2education.com/hospitals) and a 4-hour

		online module for pediatricians and gynecologists (http://www.wellstart.org/Self-Study-Module.pdf).
Breastfeeding Symposium for CMC healthcare providers	Apr 2017	A lactation consultant expert from JHI led a one-day symposium for an audience of CMC's doctors, nurses, and midwives in the NICU and maternity units. It included both practical and didactic sessions.
STEP 3. Inform all pregnant women about the benefits and management of breastfeeding.		
OB/GYN counseling on EBF	Feb 2016	The antenatal visit record indicates whether or not a patient is planning to breastfeed, but not whether counseling was provided by the physician. The medical record is available at delivery (electronic if the patient was seen at CMC, or hard copy of her prenatal visits were elsewhere).
Antenatal education on EBF by nurse educators or midwives	Feb 2016	Antenatal classes for expecting couples who plan to deliver at CMC include a 2.5-hour session on breastfeeding and the BFHI model. The classes are optional and efforts are underway to increase attendance through outreach to physicians who refer their patients. They are also promoted on CMC's website and Facebook page.
Launch of EBF promotional campaign within the hospital	May 2016	Banners, pins, bibs, and newborn hats were distributed around the hospital with messages supporting breastfeeding women.
Creation of EBF video targeted at healthcare providers to encourage breastfeeding	Jan 2017	Video produced by JHI and professional videographer.
STEP 4. Help mothers initiate breastfeeding within a half-hour of birth. (Place babies in skin-to-skin contact with their mothers immediately following birth for at least an hour. Encourage mothers to recognize when their babies are ready to breastfeed and offer help if needed.)		
Development of skin-to-skin policy	July 2016	This policy recommends uninterrupted skin-to-skin contact as soon as possible after birth and for at least one uninterrupted hour. Skin-to-skin contact is advised and performed with all mothers and babies in a stable clinical condition.

Tracking of beginning and end of skin-to-skin time	July 2016	This indicator is recorded by midwives on the newborn flow sheets (now part of CMC's electronic medical record).
STEP 5. Show mothers how to breastfeed and how to maintain lactation, even if they should be separated from their infants.		
Allocation of a lactation consultant to the maternity unit	Oct 2015	A specialist with Masters-level training is present on the wards 40 hrs/wk to provide lactation consultations, address difficult cases, and supervise the team of midwives who promote breastfeeding to patients.
Creation of a "Breastfeeding Room"	Jan 2016	This room is located in the maternity unit and provides space for patients/staff who need to breastfeed or pump, as well as space for patients to receive walk-in postnatal consultations with a nurse or midwife. Newborns with special conditions are examined by the pediatrician and the lactation consultant.
Documentation of ambulatory mother education sessions	Jan 2016	Inpatient and discharge education sessions with patients are recorded in the progress note and the "education sheet" in the electronic medical record.
STEP 6. Give newborn infants no food or drink other than breast milk, unless medically indicated.		
Revision of existing "no formula on the wards" policy	Oct 2016	This included reinforcing the hospital's compliance with the International Code of Marketing Breastmilk Substitutes and enacting a new policy to keep formula and powdered milk under lock and key. Women were still able to access formula if they declined to breastfeed after receiving support and education.
Addition of requirement of medical order by pediatrician when formula is needed	Jan 2016	Women who cannot breastfeed for medical reasons receive individual counseling. In order to receive infant formula, the pediatrician must write a medical order.
Creation of newborn flow sheets	Nov 2015 – Dec 2015	Separate from the overall medical progress notes, the newborn flow sheet is dedicated to newborn feeding and elimination. It was originally paper-based but was upgraded to an electronic version and incorporated into CMC's EMR system in Feb 2017. The flow sheets are used to calculate the EBF indicator over time.

STEP 7. Practice rooming-in – allow mothers and infants to remain together 24 hours a day.		
Development and implementation of rooming-in policy	Oct 2015	This policy includes procedures and safety precautions related to rooming-in.
Purchase of room basins	Jan 2016 – Apr 2016	Room basins in each patient's room allowed for easier implementation of the rooming-in policy.
Change name of nursery to "newborn assessment room" to promote rooming-in	Jan 2016	This room is used for infant care, which includes bathing, circumcision, and blood tests.
Tracking of rooming-in on the newborn flow sheets	May 2016	Rooming-in, or any reason for not doing so, is recorded by midwives on the newborn flow sheet.
STEP 8. Encourage breastfeeding on demand.		
Creation of in-hospital EBF indicator	Nov 2015	Tracking of EBF rates during hospital stay has been a key progress indicator for JHI's collaboration with CMC. It is part of the management dashboard, and is also used by the health promotion midwife / lactation consultant to improve practices on the ward.
Addition of LATCH score to newborn flow sheet	Nov 2015	The LATCH score documents the ability of the newborn to successfully breastfeed and is used to address any barriers to breastfeeding that may be present.
STEP 9. Give no artificial teats or pacifiers (also called dummies or soothers) to breastfeeding infants.		
Discouragement of artificial pacifiers on the ward	March 2016	The maternity team is trained to advise against the use of pacifiers and educate patients on why they shouldn't be used. In the cases where women bring pacifiers from home, it is documented in the medical record.
STEP 10. Foster the establishment of breastfeeding support groups and refer mothers to them on discharge from the hospital or clinic.		
Provision of information on breastfeeding at discharge	Nov 2015	Women receive a two-page document on breastfeeding and baby care (in Arabic and English) at discharge. They are also given information on the postnatal service with the lactation consultant (available Mon-Fri 7 am-3 pm) and the phone support line (available 24/7). There is no referral to community breastfeeding support groups.
Creation of a 24-hour phone line for breastfeeding support	Feb 2016	Three midwives are on-call 24 hours a day to answer women's questions about breastfeeding and other newborn care topics.
